# A putative biomarker signature for clinically effective AKT inhibition: correlation of *in vitro*, *in vivo* and clinical data identifies the importance of modulation of the mTORC1 pathway

**DOI:** 10.18632/oncotarget.6153

**Published:** 2015-10-19

**Authors:** Azadeh Cheraghchi-Bashi, Christine A. Parker, Ed Curry, Jean-Frederic Salazar, Hatice Gungor, Azeem Saleem, Paula Cunnea, Nona Rama, Cristian Salinas, Gordon B. Mills, Shannon R. Morris, Rakesh Kumar, Hani Gabra, Euan A. Stronach

**Affiliations:** ^1^ Ovarian Cancer Action Research Centre, Department of Surgery and Cancer, Imperial College London, Hammersmith Campus, London, UK; ^2^ GlaxoSmithKline, Clinical Imaging Centre, Hammersmith Hospital, London, UK; ^3^ Division of Experimental Medicine, Centre for Neuroscience, Imperial College, London, UK; ^4^ Department of Surgery and Cancer, Faculty of Medicine, Imperial College, London, UK; ^5^ The University of Texas MD Anderson Cancer Center, Houston, TX, USA; ^6^ GlaxoSmithKline, Oncology R&D, Research Triangle Park, NC, USA; ^7^ GlaxoSmithKline, Oncology R&D, Collegeville, PA, USA

**Keywords:** ovarian cancer, platinum resistance, biomarkers, proteomics, AKT

## Abstract

Our identification of dysregulation of the AKT pathway in ovarian cancer as a platinum resistance specific event led to a comprehensive analysis of *in vitro*, *in vivo* and clinical behaviour of the AKT inhibitor GSK2141795. Proteomic biomarker signatures correlating with effects of GSK2141795 were developed using *in vitro* and *in vivo* models, well characterised for related molecular, phenotypic and imaging endpoints. Signatures were validated in temporally paired biopsies from patients treated with GSK2141795 in a clinical study. GSK2141795 caused growth-arrest as single agent *in vitro*, enhanced cisplatin-induced apoptosis *in vitro* and reduced tumour volume in combination with platinum *in vivo*. GSK2141795 treatment *in vitro* and *in vivo* resulted in ~50-90% decrease in phospho-PRAS40 and 20-80% decrease in fluoro-deoxyglucose (FDG) uptake. Proteomic analysis of GSK2141795 *in vitro* and *in vivo* identified a signature of pathway inhibition including changes in AKT and p38 phosphorylation and total Bim, IGF1R, AR and YB1 levels. In patient biopsies, prior to treatment with GSK2141795 in a phase 1 clinical trial, this signature was predictive of post-treatment changes in the response marker CA125. Development of this signature represents an opportunity to demonstrate the clinical importance of AKT inhibition for re-sensitisation of platinum resistant ovarian cancer to platinum.

## INTRODUCTION

Epithelial ovarian cancer (EOC) is the most lethal form of gynaecological malignancy, yet it typically presents as a chemo-sensitive disease. This paradox is explained by the frequent emergence of resistance to current therapeutic regimens combining platinum compounds and taxanes [1]. Addition of further cytotoxic combinations have not produced clinical improvement [2], and thus the focus has turned to targeted therapeutics, selected on the basis of our understanding of ovarian tumour biology.

EOC is a heterogeneous group of diseases representing distinct histological subtypes including serous, endometrioid, clear cell, and mucinous. Serous ovarian cancer has been further divided into Type I and Type II tumours which represent distinct clinical and genetic entities [3]. One of the most frequently activated pathways in EOC is the PI3K/AKT oncogenic network. PI3K (~40%), KRAS (~20%) and PTEN (~5%) mutations underlie activation in Type I tumours, whereas Type II tumours exhibit frequent copy number changes and over-expression of PI3K/AKT pathway components (~46%) [4]. We showed that activation of AKT in platinum-resistant cells occurs by phosphorylation of AKT at serine 473 by DNA-dependent protein kinase (DNA-PK) [5] following DNA damage, directly linking the established role of AKT in chemo-resistance to platinum-mediated DNA damage [4, 6, 7]. We also demonstrated that inhibition of AKT or DNA-PK restores sensitivity to platinum in clinically resistant EOC cells [5]. These data suggest AKT is a strong anti-cancer target for platinum resistant disease and several AKT inhibitors have been developed and are reviewed elsewhere [6, 7]. GlaxoSmithKline has developed GSK2141795, which is a potent N-alkyl pyrazole pan-AKT inhibitor, with high affinity for all three AKT isoforms [8, 9].

The development of drugs for treatment of cancer remains a slow, expensive and high-risk process with many compounds failing due to lack of therapeutic activity, toxicity and poor pharmacokinetics. Additionally, the difficulty in translating behaviour of anti-cancer agents from *in vitro* to *in vivo*, and subsequently the clinical environment has contributed to this failure. A key area of focus in the drug discovery process is identification and validation of reliable predictive and pharmacodynamic (PD) biomarkers. PD biomarkers may include cellular, molecular, histopathological and/or imaging parameters [10, 11]. Such PD biomarkers should clearly enable efficient and scientifically-driven “Go/NoGo” decisions allowing acceleration of the drug development process.

This study was designed to evaluate the activity of the specific AKT inhibitor GSK2141795 [9] alone and in combination with platinum in platinum-resistant ovarian cancer and to guide the clinical development of AKT inhibition by identifying protein-based and imaging-based predictive and pharmacodynamic biomarkers. Significantly, we report the identification of a proteomic signature of response to AKT inhibition using *in vitro* and *in vivo* samples and subsequent validation using clinical biopsies taken before and after treatment with GSK2141795. This signature will be of use for predicting response to AKT inhibition in the clinic.

## RESULTS

### Inhibition of AKT results in growth arrest alone and apoptosis in combination with cisplatin in platinum-resistant ovarian cancer cells

**Figure 1:**
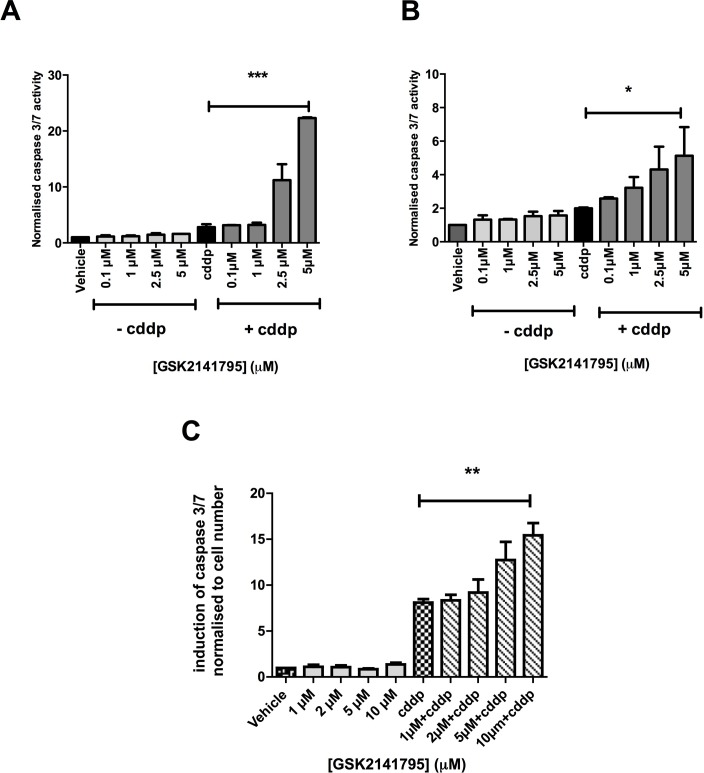
Caspase 3/7 activity in SKOV3 and PEO4 cells exposed to GSK2141795 as a single agent or in combination with cisplatin SKOV3 and PEO4 monolayers (**A.** and **B.**, respectively) and SKOV3 spheroids **C.** were pre-treated with a range of concentrations of GSK2141795, 1 hour prior to treatment with cisplatin (cddp; 25 μM). Induction of caspase 3/7 activity was assessed at 24 hours following the initiation of the treatment for the monolayers **A.** and **B.** and at 72 hours for the spheroid **C.**. Data shown are the means ± SEM of 3-4 experiments performed in triplicate. *p < 0.05, **p < 0.01, ***p < 0.001 (paired t-test).

Platinum-resistant SKOV3 cells, grown as monolayers, were treated with GSK2141795 alone or with cisplatin for 24, 48 and 72 hours. Caspase 3/7 activity was assessed as a marker of apoptosis at each time point. GSK2141795 treatment alone did not induce caspase activation, however significantly enhanced apoptosis induced by cisplatin at all time points tested (Figure 1A and Supplementary Figure S1). Similar results were obtained for platinum-resistant PEO4 ovarian cancer cell monolayers (Figure 1B) and SKOV3 spheroids (Figure 1C).

**Figure 2:**
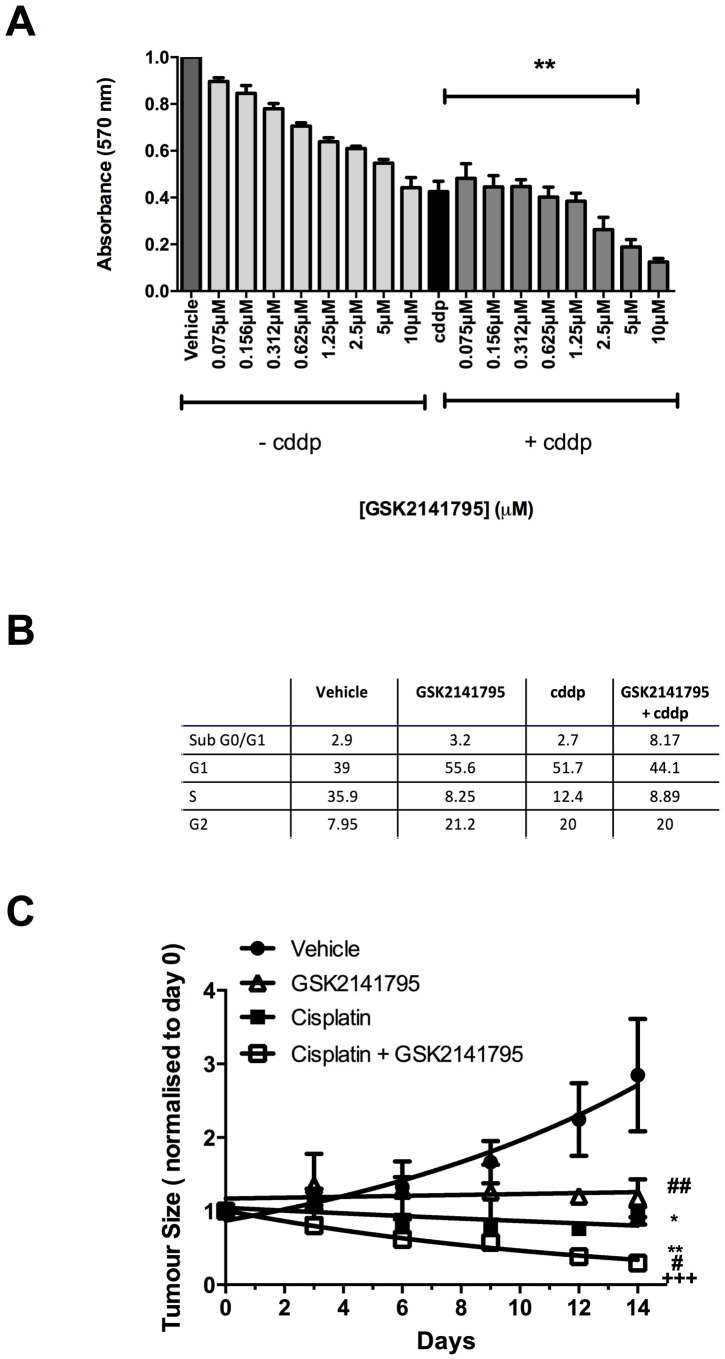
Effect of GSK2141795 either alone or in combination with cisplatin on the viability, cell cycle and tumor growth of SKOV3 cells SKOV3 cells were exposed to a range of GSK2141795 concentrations (0.075 - 10 μM) either as a single agent or in combination with cisplatin (cddp; 25 μM) for 72 hours, when cell viability was measured using MTT **A.** Cell cycle analysis of SKOV3 cells following treatment with GSK2141795 as a single agent (5 μM) or in combination with cisplatin (25 μM) for 24 hours **B.** SKOV3 tumour-bearing mice were dosed daily with GSK2141795 (30 mg/Kg; oral) or vehicle ± biweekly cisplatin (1.5 mg/Kg; intraperitonal) for 14 days **C.**. Data shown in **A.** and **B.** are the means ± SEM of 3-4 experiments performed in triplicate, and in **C.** the mean ± SEM for *n* = 8 tumours/treatment, *p < 0.05, **p < 0.01, ***p < 0.001 (paired t-test), where the symbols *, # and + represent significant differences when compared to vehicle, cisplatin and GSK2141795 data at 14 days, respectively.

Cell viability in 2D-monolayers was measured by MTT assay, which measures cellular metabolism as a surrogate of viability, under conditions identical to apoptosis experiments. SKOV3 cells treated GSK2141795 showed a dose dependent decrease in cell viability (Figure 2A). Interestingly, this was despite no increase in apoptosis detected by caspase 3/7 assay (Figure 1A), and is thus consistent with MTT changes predominantly representing growth arrest rather than apoptosis. Treatment with cisplatin alone reduced cell viability, and this was further decreased in a GSK2141795 dose-dependent manner on combination (Figure 2A: >50% reduction with cisplatin and 5μM GSK2141795 relative to cisplatin-treatment alone (*p* < 0.01)), and the half-maximal effective concentration of GSK2141795 in the combination treatment was 3μM. Cell cycle analysis by flow cytometry indicated G1 and G2 arrest in SKOV3 cells treated with GSK2141795 alone, but no increase in apoptosis, consistent with caspase 3/7 and MTT assay data (Figure 2B). Co-treatment with GSK2141795 and cisplatin increased sub-G0/G1 fraction compared to either drug treatment alone, consistent with caspase activation data (Figure 2B and Supplementary Figure S2).

The combinatorial effect of varying concentrations of cisplatin and GSK2141795 *in vitro* was assessed by isobologram analysis and indicated synergy in both SKOV3 and PEO4 cells (Supplementary Figure S3).

Tumour growth rates of SKOV3 tumour-bearing mice were assessed following daily dosing with vehicle or GSK2141795 either alone or in combination with cisplatin. Treatment with cisplatin alone caused a significant decrease in tumour size compared to vehicle-treated animals at day 14 (*p* < 0.05; Figure 2C). When GSK2141795 and cisplatin were used in combination, the tumour growth rates were further decreased compared to cisplatin only treated animals (*p* < 0.01; Figure 2C).

Concentrations of GSK2141795 in tumour and blood taken from *in vivo* SKOV3 xenograft time-course studies were determined. GSK2141795 accumulated in the SKOV3 xenograft tumours during the first 24 hours of treatment, with 12.5 hour half-life of the drug in the tumour (Supplementary Figure S4). In comparison, GSK2141795 was elevated in the blood for 60 minutes following oral dosing, decreasing rapidly to low levels for the remainder of the time-course (Supplementary Figure S4).

### AKT inhibition via GSK2141795 decreases phosphorylation of the pharmacodynamic biomarker PRAS40 at Thr246 in a dose-dependent manner

**Figure 3:**
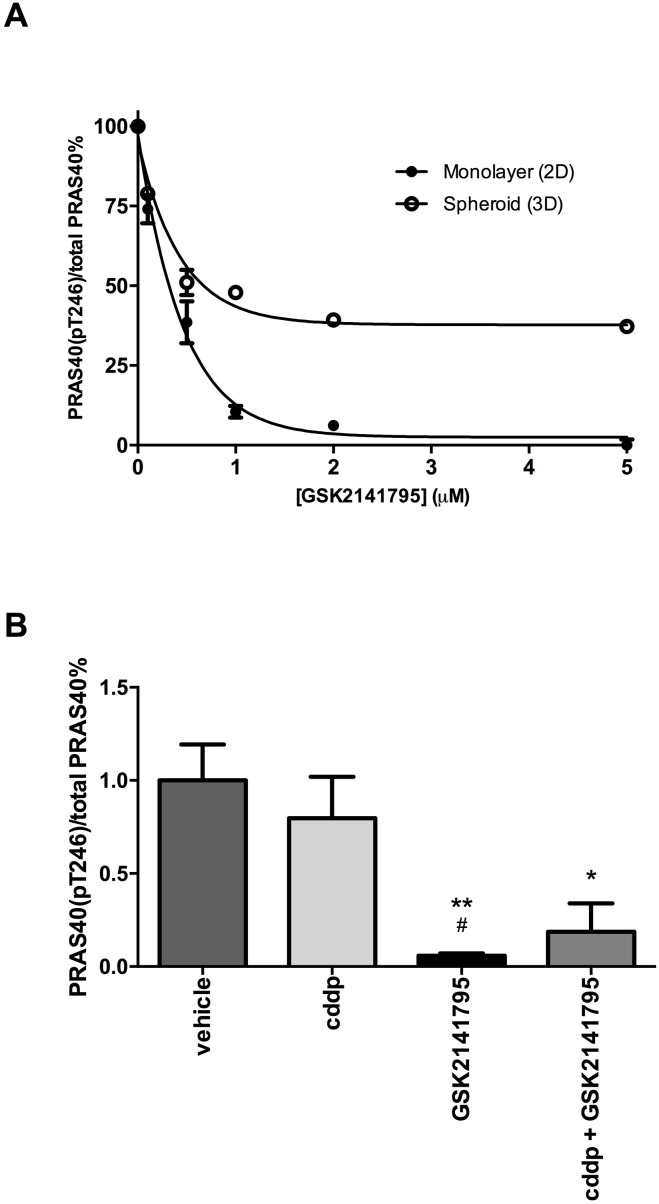
Inhibition of PRAS40 phosphorylation by GSK2141795 in SKOV3 monolayers, spheroids and xenografts Protein concentration of phospho-PRAS40 (Thr246) and total PRAS40 was determined by enzyme-linked immunosorbent assay (ELISA) after 72hr treatment of SKOV3 cells with a range of concentrations of GSK2141795 (0.01 - 5 μM) in both monolayers and spheroids **A.**. GSK2141795 (30 mg/Kg) abolished phosphorylation of PRAS40 at Thr246, both as single agent and in combination with cisplatin (cddp), in SKOV3 tumour xenografts following 14 days of treatment **B.**. Data are presented as a phospho-PRAS40 / total PRAS40 decrease relative to untreated or vehicle treated samples. Data shown in **A.** are the means ± SEM of *n* = 2 experiments performed in triplicate, and in **B.** the mean ± SEM for *n* = 5 animals (tumours) / treatment. *p < 0.05 and **p < 0.01, where the symbols * and # represent significant differences when compared to vehicle and cisplatin data, respectively (unpaired t-test, two tailed).

To confirm the ability of GSK2141795 to inhibit AKT signalling in SKOV3 cells, lysates from 2D monolayers and 3D spheroids were treated with GSK2141795 and analysed for phosphorylation of its downstream substrate PRAS40. Treatment with GSK2141795 resulted in a concentration-dependent reduction in the ratio of phosphorylated PRAS40 (Thr246) to total PRAS40 in both SKOV3 monolayers and 3D spheroids (Figure 3A). Phosphorylation of PRAS40 at Thr246 was reduced to < 1% pretreatment level in SKOV3 monolayers treated with GSK2141795 (5μM) for both 72 hours (Figure 3A) and 48 hours (Supplementary Figure S5). Reduction in phosphorylation was only partial (~50% of the pretreatment level) in the 3D model at the highest concentration, perhaps reflecting partial penetration of the compound into the 3D spheroids (Figure 3A). Additionally, levels of phosphorylated PRAS40 were significantly reduced in SKOV3 xenografts following treatment with either GSK2141795 as a single agent (*p* < 0.01) or in combination with cisplatin (*p* < 0.05; Figure 3B). This effect was not observed when using cisplatin as a single agent in these xenografts.

### AKT inhibition by GSK2141795 decreases [^3^H]FDG uptake in 2D, 3D spheroids and tumour xenograft models of ovarian cancer

**Figure 4:**
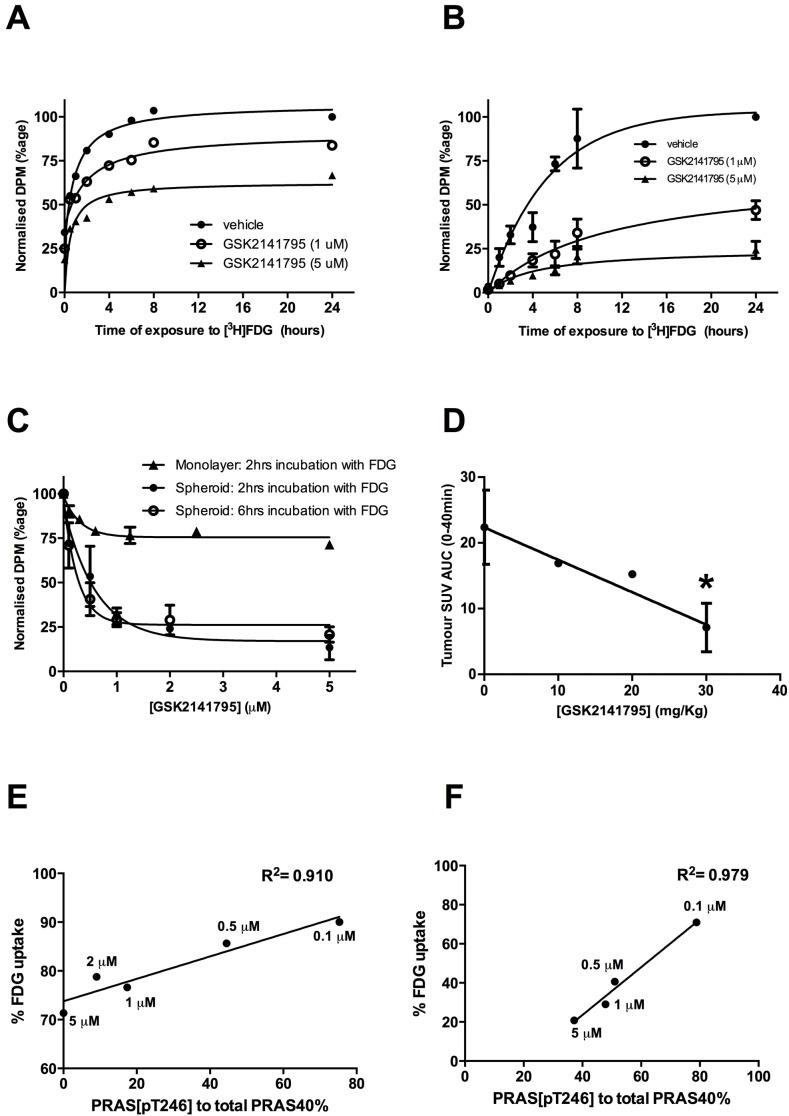
Effect of GSK2141795 on FDG uptake in SKOV3 monolayer, spheroids and mouse xenografts Time-course of [^3^H]FDG uptake in SKOV3 monolayers (*N* = 3) **A.** and spheroids (*N* = 3, experiments performed in triplicate ± SEM) **B.** pre-incubated with either vehicle or GSK2141795 (1 and 5 μM) for 48 hours; Dose-dependent effect of GSK2141795 on the uptake of [^3^H]FDG into SKOV3 monolayers following 2 hours incubation with [^3^H]FDG; SKOV3 spheroids following 2 and 6 hours incubation with [^3^H]FDG (*N* = 3, experiments performed in triplicate ± SEM) **C.**; and uptake of [^18^F]FDG into tumours of SKOV3 xenografted mice following 5 hours drug treatment (10 - 30 mg/Kg; *n* = 3 per dose except for 20 mg/Kg where *n* = 1), *p < 0.05 compared to vehicle treated animals (unpaired t-test) **D.**. Pearson correlation between expression levels of phospho-PRAS40 (Thr246) and [^3^H]FDG uptake into SKOV3 monolayers **E.** and spheroids **F.** following treatment with GSK2141795. SKOV3 monolayers and spheroids were treated with varying concentrations of GSK2141795 for 48 hours. Phospho-PRAS40 (Thr246) and [^3^H]FDG uptake were analysed as described in the methods section. Abbreviations: DPM = decays per minute; SUV = standardised uptake values; AUC = area under the curve.

Uptake of radiolabelled FDG into SKOV3 monolayers and spheroids, treated with GSK2141795 for 24 and 48 hours respectively, was measured over a 24-hour time-course. [^3^H]FDG uptake into vehicle treated monolayers and spheroids reached a plateau at approximately 6 and 10 hours, respectively (t½ = 0.52 hours (monolayers) and 3.04 hours (spheroids); Figure 4A and 4B). Additionally, these *in vitro* time-course studies demonstrated dose-dependent decrease in FDG uptake upon treatment with GSK2141795 (t½ = 0.41 and 0.53 hours for 1 and 5 μM, respectively (monolayers); and 5.79 and 4.30 hours for 1 and 5 μM, respectively (spheroids)) (Figure 4A and 4B). In addition, SKOV3 tumour bearing mice were treated with GSK2141795 for 1 to 72 hours prior to [^18^F]FDG administration. Uptake of [^18^F]FDG into SKOV3 tumours decreased in a time-dependent manner (t½ = 12 hours) following oral administration of GSK2141795 (30mg/kg; Supplementary Figure S4A); which results in blood and tumour C_max_ >5μM GSK2141795 (Supplementary Figure S4B-D).

Although maximal uptake of [^3^H]FDG into SKOV3 monolayers and spheroids occurred at approximately 6 and 10 hours respectively, it is not feasible to use these time points when scanning patients with [^18^F]FDG in the clinic. Therefore, dose-response studies utilising GSK2141795 were repeated in SKOV3 monolayers at 2 hours (optimum time for clinical scanning; Figure 4C) and spheroids at both 2 and 6 hours (6 hours = optimum time for incubation of the tumours with radio-ligand; Figure 4C) to allow comparison. A dose-dependent decrease in [^3^H]FDG uptake was observed in the SKOV3 monolayers (ED_50_ = 0.22 μM; Figure 4C) which formed a plateau at ~1 μM GSK2141795. A greater concentration-dependent effect of GSK2141795 was observed in the SKOV3 spheroids at both the 2 and 6 hour time-points, again reaching a plateau at ~1 μM, yielding similar ED_50_ values (ED_50_ = 0.42 μM (2 hours) and 0.18 μM (6 hours); Figure 4C). *In vivo* analysis of the effect of GSK2141795 on [^18^F]FDG uptake into SKOV3 xenografts indicated reduced [^18^F]FDG signal compared to vehicle reaching a maximum of 68% at the highest dose of 30 mg/kg of GSK2141795 (*p* < 0.05) used in this study (Figure 4D).

The relationship between decreases in FDG uptake in cells and changes in downstream markers of AKT pathway inhibition (phospho-PRAS40/total PRAS40) induced by GSK2141795 were evaluated in SKOV3 monolayers and spheroids. These comparisons revealed a strong positive correlation between the two parameters over the range of GSK2141795 concentrations in both the SKOV3 monolayers (R^2^ = 0.910; Figure 4E) and spheroids (R^2^ = 0.979; Figure 4F).

### Identification of a proteomic signature of AKT pathway inhibition by GSK2141795 by reverse phase protein array

A proteomic signature of AKT pathway inhibition by GSK2141795 was identified through RPPA analysis of SKOV3 tumours grown *in vivo,* SKOV3 monolayers *in vitro* and PEO4 monolayers *in vitro.* Consistent effects of treatment with GSK2141795 across the range of environments studied (each compared to a DMSO control) were identified using a Rank Product meta-analysis. This analysis enabled assessment of the statistical significance of treatment effect on protein expression across the set of studies, and the resulting signature is given in Table 1.

**Table 1:** Proteomic signature of protein level changes following GSK2141795 treatment across xenografts and cell lines as described in Materials and Methods

**Proteins increased on GSK2141795 treatment**	**FoldChange**	**P-value**	**Adj P-value**
Akt_pS473	1.327855	0.000100	0.003125
Akt_pT308	2.181444	0.000100	0.003125
p38_pT180_Y182	1.144112	0.000100	0.003125
STAT5_alpha	1.304476	0.000100	0.003125
Bim	1.248445	0.001990	0.049760
IGF_1R_beta	1.144254	0.004482	0.093367
YAP_pS127	1.148339	0.006125	0.109371
GSK3_alpha_beta_pS21_S9	0.421782	0.008931	0.139550
MAPK_pT202_Y204	0.968154	0.014187	0.197044
AR	1.081206	0.017242	0.215530
YB_1	1.177568	0.031214	0.354700
**Proteins decreased on GSK2141795 treatment**	**FoldChange**	**P-value**	**Adj P-value**
Cyclin_B1	0.672057	0.000100	0.003950
S6_pS235_S236	0.137526	0.000100	0.003950
S6_pS240_S244	0.194702	0.000100	0.003950
Caspase_7_cleaved_D198	0.797813	0.000126	0.003950
Rb_pS807_S811	0.709457	0.000175	0.004380
EGFR	0.799100	0.000595	0.012400
53BP1	0.875146	0.001762	0.031471
IGFBP2	0.896407	0.002767	0.043238
NF2	0.821243	0.005116	0.071056
4E.BP1_pS65	0.713764	0.010503	0.131290
Tuberin	0.788187	0.011787	0.132717
ACC1	0.864443	0.012741	0.132717
EGFR_pY992	0.961440	0.021906	0.210638
VEGFR2	0.897841	0.029226	0.248513
Fibronectin	0.807220	0.030147	0.248513
PRAS40_pT246	0.668444	0.031810	0.248513
ACC_pS79	0.858526	0.035321	0.259712

**A cut-off of p<0.05 in the RankProd meta-analysis was applied.**

From this proteomic signature, a subset of pathway alterations showing a similar change upon GSK2141795 treatment in at least 4 of 5 experimental groups (studies) was identified (given in Supplementary Table S2). These alterations reflect the *most* consistent of the changes across all experimental models. They included an increase in phosphorylation of AKT at T308 and S473, increased phosphorylation of p38 MAPK and increases in total AR, Bim, IGF1R and YB1. Decreases in phosphorylation of S6, Rb, ACC1, 4EBP1 and PRAS40 were observed as well as decreases in total NF2 and Tuberin.

### Proteomics-based pharmacodynamic biomarkers in clinical administration of drug

**Figure 5:**
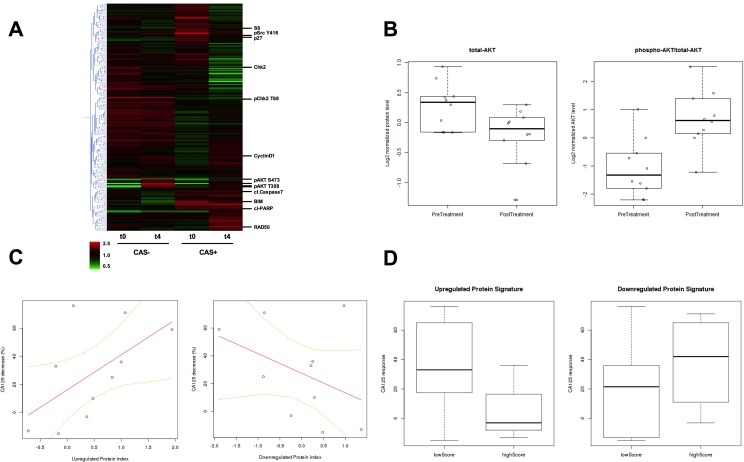
**A.** Hierarchical clustering heatmap of protein expression detected in RPPA from pre-treatment and W4 on-treatment biopsies. CAS+: patient group with clinical activity signal, and CAS-: patient group with no clinical activity signal are indicated. t0: pre-treatment and t4: W4 on-treatment biopsy samples. The expression level for each protein from each patient at a given time point in the CAS or non-CAS population is averaged and displayed in the heatmap, with data for each protein median centered. Red indicates an increase in expression and green represents a decrease in expression with respect to the median. Examples of significant proteins identified in the analysis are indicated individually. **B.** Comparison of the average levels of total AKT and the ratio of phospho-AKT/total-AKT between pre-treatment biopsies (*n* = 12) and the biopsies taken following 4 weeks of treatment with GSK2141795 (*n* = 10) for all patients. Total AKT decrease student's *t*-test *p* = 0.02; phospho-AKT/total-AKT increase student's *t*-test *p* = 0.006 **C.** Correlation between clinical response to administration of GSK2141795 and pharmacologically-induced change in protein expression profile for AKT-inhibition signature. Percentage decrease in patients' CA125 levels is plotted against the difference in AKT-inhibition proteomic signature z-scores between pre-treatment biopsy and the biopsy taken following 4 weeks of treatment with GSK2141795 (*n* = 10 pairs) for the up- and down-regulated protein signatures (Table 1). T distribution of Pearson correlation coefficient gives *p* = 0.082 for up-regulated protein signature. **D.** Comparison of treatment-induced decrease in CA125 (as a percentage of pre-treatment level) following 4 weeks of treatment with GSK2141795 in patients with low versus high pre-treatment levels of the up- and down-regulated AKT-inhibition proteomic signatures (Table 1). T distribution of Pearson correlation coefficient between CA125 decrease and up-regulated proteomic signature score gives *p* = 0.041. CAS: clinical activity signal. CAS+ >30% CA125 decrease. CAS- < 30% CA125 decrease.

From each of 10 patients participating in a Phase I clinical trial for GSK2141795, cell lysates from one biopsy taken prior to treatment and one biopsy taken following 4 weeks (W4) of treatment with the drug were profiled using Reverse Phase Proteomic Arrays (as for the *in vitro* and xenograft studies) [8]. Protein levels in paired biopsies (pre-dose and W4 post-dose) were evaluated by RPPA for evidence of target inhibition by GSK2141795, and to obtain insight into how signal transduction networks responded and adapted to AKT inhibition. Hierarchical clustering analysis was carried out on RPPA data from pre and post treatment biopsies using a cutoff of 30% maximal decrease in CA125 as a definition of clinical activity (CAS: clinical activity signal. CAS+ >30% CA125 decrease. CAS- < 30% CA125 decrease). Clustering heatmaps indicate profound changes in signaling behaviour following GSK2141795 treatment in the patient group with clinical activity (>30% CA125 decrease) (Figure 5A). Conversely in the patient group without clinical activity, very little effect is seen in the RPPA data beyond alterations in phospho-AKT itself, indicating drug activity but no downstream molecular effect (Figure 5A). As shown in Figure 5B, both total AKT (decreased; *p* = 0.02) and the phospho-AKT/total AKT ratio (increased; *p* = 0.006) showed significant fold-changes in expression levels at W4 compared to baseline by RPPA in all patients regardless of CA125 response, consistent with pharmacodynamic target engagement, thus providing clinical validity of the RPPA analysis. The change in phospho-AKT levels at W4 correlated with changes seen previously by immuno-histochemistry [8].

### Pharmacologically-induced changes in proteomic signature of AKT inhibition correlate with therapeutic response

Using the experimentally-derived proteomic signature of AKT inhibition via treatment with GSK2141795 (Table 1), we calculated signature scores to reflect the degree of AKT pathway modulation in this series of biopsies taken from patients undergoing treatment with GSK2141795 in the clinic. Clinical response in patients receiving the drug was inferred from the percentage drop in levels of the ovarian cancer marker CA125. Interestingly, the difference in signature scores between pre-treatment and post-treatment biopsies for each patient were correlated to the corresponding decrease in CA125 levels, as shown in Figure 5C. It can be seen that as expression levels of the *in vitro* GSK2141795-upregulated proteins (see Table 1) systematically increase following clinical administration of the drug, there is a trend towards the patient's CA125 levels decreasing correspondingly (*p* = 0.082). A similar effect is observed for a decrease in expression of the *in vitro* GSK2141795-downregulated proteins (*p* = ns). This indicates that successful pharmacological modulation of the AKT pathway in ovarian tumours is reflected by a decrease in the patient's overall tumour burden.

### Pre-treatment expression levels of AKT-inhibition signature proteins are predictive of clinical response

The clinical activity in patients receiving GSK2141795, as indicated by the percentage decrease in CA125 levels, was significantly greater in patients with low pre-treatment levels of the *in vitro* GSK2141795-upregulated proteins (see Table 1) than it was for the patients with high pre-treatment levels of these proteins. Figure 5D (left panel) shows the distributions of CA125 decrease for the low-expressing group (consisting of patients for which z-scores for the GSK2141795-upregulated signature were less than zero in pre-treatment biopsies, meaning the tumours were expressing the proteins at relatively low levels), and the high-expressing group (the remaining patients, which all had z-scores greater than zero). The correlation between low pre-treatment expression of the GSK2141795-upregulated proteins and the treatment-induced reduction in CA125 was statistically significant (*p* = 0.041), despite the small number of patients with paired biopsies (*n* = 10). A similar effect, although not statistically significant, is also observed in which response tends to be better in patients with high pre-treatment levels of the GSK2141795-downregulated proteins (Figure 5D (right panel)). These results suggest that it is possible to predict the clinical benefit a patient may gain from treatment with AKT inhibitors, based on the proteomic signatures derived from our experiments involving *in vitro* and *in vivo* model systems.

## DISCUSSION

The AKT pathway has been shown to have a role in chemotherapy resistance in a variety of situations [12]. This study describes the first demonstration of the ability of the ATP competitive, pan-AKT inhibitor, GSK2141795 [9], to restore platinum-sensitivity to cells that developed platinum resistance in patients. In addition, we have demonstrated the utility of FDG-PET imaging as a pharmacodynamic marker via correlating changes in glucose metabolism (as measured by changes in FDG uptake) with changes in downstream biomarkers (phospho-PRAS40) of AKT inhibition and have importantly shown that proteomic signatures derived from our *in vitro* and *in vivo* experiments are predictive of patient PD and CA125 response to GSK2141795 in the clinic.

GSK2141795 as a single agent did not increase levels of caspase 3/7 activity in platinum-resistant cell lines, despite inhibiting AKT enzymatic activity, as inferred by decrease in phosphorylation of PRAS40. The drug did however cause growth arrest as a single agent, as indicated by cell cycle analysis and MTT assay. This observation indicates that increases in caspase 3/7 activity, indicative of increased apoptosis, observed when GSK2141795 was combined with cisplatin were due to synergy between the two agents, a finding which was confirmed by subsequent isobologram analysis. This has important implications for the use of AKT inhibitors suggesting that synergistic combinations with drugs that enhance apoptosis would be predicted to be more clinically efficacious than single agent treatment, which may only cause temporary tumour growth arrest.

Given these data and the clinical unmet need of ovarian cancer patients with platinum-resistant disease, combining GSK2141795 with platinum-based agents in the clinic is imperative. However, combining anti-cancer agents in the clinic is not always straightforward; doses of drugs used in combination often have to be reduced compared to when they are given as monotherapies. Yet it is not always clear that the reduced dose will have the same effects on the target pathway, i.e. what is the minimal level of pathway inhibition required to maintain efficacy. Ideally, quantification of pathway inhibition would be done with serial biopsies of a patient's tumour before and after drug treatment, in order to evaluate levels of phosphorylated AKT substrates. Although paired biopsies were achieved in the trial described here, clinical implementation of this approach can be challenging due to the lack of infrastructure at most centres for collection and processing of biopsies, the invasive nature of repeat biopsies or lack of biopsiable tumours in some patients. Therefore having a way to measure pathway inhibition in a non-invasive fashion would be of great benefit to this process.

The preclinical FDG-PET studies described in this manuscript suggest that FDG-PET may be a useful pharmacodynamic biomarker for reflecting the effects of GSK2141795 on tumour glucose metabolism in the clinic. Identical FDG-PET studies were performed using SKOV3 cells as (1) monolayers, (2) spheroids and (3) *in vivo* xenografts. This allowed for comparison of the data obtained with the three models in order to determine the inter-predictability of each model and the relationship between changes in phospho-PRAS40 and FDG uptake in various models. Our data shows that although phospho-PRAS40 levels were decreased by >50% in all 3 model systems, only spheroids and *in vivo* xenografts showed >50% decrease in FDG uptake, suggesting that spheroids may be preferable to monolayer cultures as an *in vitro* representation of *in vivo* physiology. Spheroids studies also demonstrated a concentration-dependent decrease in FDG signal following GSK2141795 administration, which was reproduced in xenograft studies. These data, coupled with the PK data obtained from xenograft studies, predict that concentrations ≥1 μM GSK2141795 would be required achieve maximal decrease in phospho-PRAS40 and FDG uptake.

In a phase I dose escalation study of GSK2141795 in cancer patients, a mean C_max_ of 398 ng/mL (0.93 μM) was observed at the maximally tolerated dose of 75 mg GSK2141795 once daily [13]. Separately, we reported, in a PK/PD phase I study, an inverse relationship between maximum GSK2141795 plasma concentrations with the maximum decrease in [^18^F]FDG uptake in patient tumours [8], i.e. as concentrations of GSK2141795 increased, FDG-uptake decreased. The lack of a clear dose response relationship in the latter study was likely due to a narrow dose range tested (25-75 mg, daily) coupled with large inter-patient variability in the pharmacokinetics of GSK2141795. Maximum GSK2141795 plasma concentrations observed in the clinical study ranged from approximately 300-800 ng/mL (0.70-1.86 μM), with >40% decrease in [18F]FDG uptake in 5 out of 6 patients achieving >1 μM Cmax on week 4 [8]. The preclinical data reported in this manuscript are in agreement with the clinical observations that AKT inhibition with GSK2141795 requires ≥1 μM concentration for optimal decreases in [^18^F]FDG uptake.

Protein level analysis of tumour xenografts and cell monolayers following treatment with GSK2141795 revealed several consistent alterations within the AKT pathway. Of particular note AKT phosphorylation at S473 and T308 were increased following dosing of tumour xenografts *in vivo* and in two different cell line models *in vitro* indicating these may represent stable molecular biomarkers of AKT inhibition by a catalytic domain inhibitor. Although counter-intuitive at face value the increase in AKT phosphorylation following treatment with an AKT kinase domain inhibitor has been reported previously [14-18]. Two main hypotheses prevail to explain this. Firstly inhibition of mTOR signalling downstream of AKT may alleviate a negative feedback loop exerted through p70-S6K/IRS-1 mediated inhibition of PI3K [15, 17]. Secondly, the occupancy of the ATP binding domain of AKT by an ATP competitive inhibitor stabilises the structure of AKT such that access to negative regulatory phosphatases is restricted [14, 16]. Experiments using catalytically inactive mutants of AKT indicated that downstream activity is not required for inhibitor-induced hyper-phosphorylation, hence favouring the second hypothesis [18]. In addition to AKT hyper-phosphorylation upon treatment with GSK2141795 we also saw increased phosphorylation of p38 MAPK and increases in total AR, Bim, IGF1R and YB-1 consistently across the independent RPPA datasets studied suggesting these as relatively robust markers of AKT pathway inhibition. The androgen receptor is phosphorylated at serine 210 by AKT, which has been reported to supress AR transactivation and transcription of AR target genes including p21 and AR itself [19]. Pro-apoptotic Bim is transcriptionally regulated by forkhead family of transcription factors, which in turn are negatively regulated by AKT-dependent phosphorylation hence Bim upregulation on AKT inhibition is indicative of on target effects of GSK2141795 and also a functional indicator of apoptotic priming [20]. Interestingly however, despite upregulation of Bim and phosphorylation of p38-MAPK following AKT inhibition, data in Figures 1 and S1 indicate that caspase activation and hence apoptosis does not occur on single agent AKT inhibition. We hypothesise that p38-MAPK phosphorylation and Bim upregulation primes the cells for apoptosis, which then occurs only following an apoptotic stimulus such as platinum treatment (Figure 1). YB-1 is an oncogenic transcription and translation factor, regulated by direct phosphorylation by AKT. At high levels it blocks protein translation and inhibits proliferation whereas AKT phosphorylation has been proposed to disable this inhibitory activity allowing translation of oncogenic proteins [21-23]. Yan *et al* reported AKT pathway analysis in preclinical (breast cancer) and clinical samples (phase 1; solid tumours) treated with the ATP-competitive AKT inhibitor GDC-0068. Consistent with our data they observed increases in pAKT and decreases in pPRAS40 and pS6 on AKT inhibitor treatment [24]. They also reported a compensatory feedback activation of ERK and HER3, which was not observed here, possible reflecting the different biology of the cancer types studied.

Importantly we showed that the significant changes upon GSK2141795 treatment *in vitro* and *in vivo* were also seen in paired clinical biopsies taken before and four weeks after GSK2141795 treatment on clinical trial and that the detection of these alterations was correlated with the extent decrease in CA125 in this small patient cohort, as a surrogate of clinical anti-tumour activity. Perhaps more importantly we also showed that the pre-treatment levels of “upregulated signature” proteins were significantly predictive of CA125 response to GSK2141795. Surprisingly we found that low levels of pAKT in pre-treatment biopsies was part of the predictive signature of response. This indicates that pAKT levels must be below a certain threshold for treatment to be effective ie that AKT inhibitor treatment is not 100% efficient and in patients with robust baseline activity, sufficient pathway down-regulation to produce 30% CA125 decrease may not be achieved. Pathway signatures identified here warrant confirmation in larger future clinical studies and to consider their utility in predicting response in combination with platinum containing chemotherapy.

To conclude, data reported in this manuscript demonstrate that the pan-AKT inhibitor, GSK2141795, modulates multiple components of AKT signalling in platinum-resistant ovarian cancer cells, most notably components of the mTOR pathway, that [^18^F]FDG uptake could be used as a non-invasive pharmacodynamic marker and that levels of AKT pathway proteins identified in pre-clinical models, are able to predict CA125 response in platinum resistant ovarian cancer patients treated with the AKT inhibitor GSK2141795. This data suggests that proteomic signature can be used to stratify patient selection and that [^18^F]FDG can be used to detect pharmacodynamic tumour response to AKT inhibition. These data have significant implication for clinical implementation of targeted therapy using AKT inhibitor strategies.

## MATERIALS AND METHODS

### Materials

The AKT inhibitor, GSK2141795 [9], was synthesized at GlaxoSmithKline. Cisplatin was obtained from Hammersmith Hospital's pharmacy. [^3^H]FDG (Specific Activity = 222 GBq/mmol; 37 MBq/ml) was obtained from American Radiolabelled Chemicals Inc., St Louis, USA and [^18^F]FDG (500 MBq) was obtained from PETNET UK. All other chemicals and reagents were of the highest grade possible. The high-grade serous ovarian cancer cell line PEO4 (cisplatin IC50 = 11.6μM [25]), derived directly from patient ascites after platinum-resistant relapse, was obtained from Dr. Simon Langdon (Edinburgh, UK). PEO4 cells have been shown previously by us to activate AKT pathway in response to cisplatin treatment and to be sensitized to platinum by AKT inhibition [5]. The ovarian carcinoma cell line SKOV3 (cisplatin IC50 = 28.8μM), which harbours a PIK3CA activating mutation (COSMIC ID: 1070900) and stably expresses pAKT [5], was obtained from ECACC.

### Flow cytometry analysis

Cells were treated with GSK2141795 (1μM or 5μM) and/or cisplatin (25μM). For combination treatment, cells were pre-treated with GSK2141795 1 hour prior to addition of cisplatin. Twenty-four hours following incubation, cells were harvested with 0.25% trypsin. Floating cells were combined with trypsinised cells prior to centrifugation. Cells (100,000) were fixed in 70% ethanol overnight at −20°C, followed by PBS wash and incubation with 0.05mg/ml propidium iodide (PI), 0.2mg/ml RNase A in PBS for 30 minutes, 4°C in the dark. Samples were analysed with a FACScalibur flow cytometer (Becton Dickinson, UK). Ten thousand threshold events per sample were collected and analysed on the basis of their FL2 fluorescence (excitation at 488nm/emission 585nm). Data were analysed using Flowjo software (Tree Star. Inc. USA).

### Phospho-PRAS40 and PRAS40 enzyme-linked immunosorbent assays (ELISA)

Levels of phosphorylated and non-phosphorylated proline-rich AKT substrate of 40kDa (phospho-PRAS40 (pT246) and total PRAS40) were assessed using PRAS40 ELISA kits (Invitrogen, Camarillo, USA, KHO0421 and KHO0411 respectively). SKOV3 monolayers or spheroids were treated with increasing concentrations of GSK2141795 (0.01 - 5μM) for 48 hours. Following treatment, cells were lysed in extraction buffer (Invitrogen, Camarillo, USA, FNN0011) and samples diluted 1 in 50 in diluent buffer. On completion of the assay, absorbance measured at 450nm. Phospho-PRAS40 levels were normalised to the total PRAS40 concentration.

### *In vitro* radioligand binding studies

For time-course studies, SKOV3 monolayers and spheroids were treated with two concentrations of GSK2141795 (1 and 5μM) for 24 and 48 hours respectively and incubated at 37°C, 5% CO_2_. Shorter exposure to GSK2141795 was considered for 2D-models due to the greater growth inhibitory effect of GSK2141795 in monolayers. Twenty four hours into the incubation, SKOV3 monolayers and spheroids were incubated with medium (0.5 ml per well) containing [^3^H]FDG (296 kBq) for 0-24 hours as indicated. For dose-response studies, SKOV3 monolayers and spheroids were treated with a range of concentrations of GSK2141795 (0.001-5μM; monolayers = 24hours; spheroids = 48hours). Monolayers and spheroids were incubated at 37°C, 5% CO_2_. Two or six hours prior to the end of incubation, monolayers and spheroids were incubated with 0.5ml medium per well containing [^3^H]FDG (296kBq). Following assay, monolayers and spheroids were washed twice with PBS and solubilised in 0.1M NaOH at 37°C overnight prior to transfer to 5ml tubes and addition of scintillation fluid (4ml; Packard Ultima Gold MV). Bound radioactivity was determined by liquid scintillation counting (Perkin Elmer, TriCarb2900). For each spheroids, 100μl of final wash was measured as background. Data were analysed by iterative non-linear regression curve fitting procedures in Prism v5.0 (GraphPad Software, San Diego, California, USA) with each experiment analysed individually.

### *In vivo* xenograft imaging studies

All animal work described was performed in accordance with the United Kingdom's ‘Guidance on the Operation of Animals’ (Scientific Procedures) Act 1986. Mice (female, Crl: NU/NU-Foxn-1, 25 - 30 g) were supplied by Charles River, UK. Subcutaneous xenografts were generated via injection of ~10million SKOV3 cells into the flank of NU/NU-Foxn-1 mice and tumours grown to approximately 100mm^3^. For tumour growth studies, mice bearing SKOV3 xenografts were randomly grouped into 4 treatment groups (5 animals per group), receiving either vehicle (20% PEG/1% DMSO), cisplatin (1.5mg/kg), single agent GSK2141795 (30mg/kg) or combination of GSK2141795 and cisplatin. Animals received GSK2141795 daily by oral gavage and cisplatin twice weekly via intraperitoneal (ip) injection. Tumour sizes were measured every other day for 14 days and growth curves generated for each group. For time-course studies, following overnight fasting mice were treated with GSK2141795 (30mg/kg) for 1-72hrs, as indicated, prior to [^18^F]FDG administration (~8MBq) via a jugular vein cannula. For dose-response studies, following overnight fasting, mice received either vehicle (20%PEG / 1%DMSO) or GSK2141795 (10, 20 or 30mg/kg) by oral gavage. At 5hours post-treatment, animals were administered [^18^F]FDG (~8 MBq) via a jugular vein cannula. All animals were scanned on a dedicated small animal PET scanner (Inveon PET/CT module, Siemens Molecular Imaging Inc. UK). Animals were kept under isofluorane anaesthesia throughout imaging. Dynamic emission scans were acquired in list mode format over 60min. Standardised uptake values (SUV) of radioactivity were calculated for the tumour region of interest (ROI) for each image of a dynamic series using the formula:

$${\text{SUVmean}}_{\left(30–60\min\right)}=\frac{\text{Tissue radioactivity concentration at time}(\mathrm{Ct})}{\text{Injected dose}(\text{at}\;t_{0})\mathrm{Bq})/\text{body weight}(g)}$$

### Reverse phase protein array (RPPA)

SKOV3 and PEO4 cells were treated with DMSO (vehicle) or 5μM GSK2141795 *in vitro* at timepoints indicated. Cells were washed twice with cold PBS and lysed using 100μL RPPA lysis buffer (1% Triton X-100, 50mM HEPES, pH 7.4, 150mM NaCl, 1.5mM MgCl_2_, 1mM EGTA, 100mM NaF, 10mM Na pyrophosphate, 10% glycerol containing freshly added protease (Roche Applied Science) and phosphatase inhibitors (VWR)) on 6-well plates. Tumours recovered from *in vivo* experiments were crushed in 100μl cold RPPA lysis buffer using an electric pellet pestle (Sigma). Lysates were centrifuged at 14000 rpm at 4°C for 15 minutes following incubation on ice for 30 minutes. Supernatants were collected and pellets discarded. Protein concentration was determined by BCA assay. Additional lysates were derived from 18 gauge core biopsies taken at screening and at week 4 from 12 patients on phase 1b trial of single agent GSK2141795 (trial number NCT01266954; ClinicalTrials.gov) [8]. Details are in Supplementary Table 1. Lysates were shipped to MD Anderson Cancer Centre and analysed by RPPA as described previously [26]. RPPA data was normalised as described [27] and analysed using Genespring GX7 (Agilent) and R with Bioconductor. Full RPPA datasets are available as Supplementary Tables S3 (*in vitro/in vivo*) and S4 (clinical).

### Identification of proteomic pharmacodynamic signature

A proteomic pharmacodynamic biomarker signature was constructed by identifying proteins with significant protein expression level changes following GSK2141795 treatment, across xenografts and cell lines. Statistical significance for each protein was evaluated using Rank Product meta-analysis [28] as implemented in the R package *RankProd*, This analysis takes into account the effects in each individual study, with each cell line treated as a separate study, 0.5hrs and 8hrs treated as separate studies, and the xenografts treated as one (separate) study. Proteins with a meta-analysis significance estimate of p < 0.05 were selected to construct the proteomic PD signature (Table 1).

### Evaluation of proteomic signature levels in clinical biopsies

The proteins constituting the pharmacodynamic signature derived from *in vitro* and *in vivo* assays were mapped to those in the RPPA profiling of biopsies taken from patients undergoing treatment with GSK2141795 as part of a phase I clinical trial (trial number: NCT01266954). AKT pathway modulation scores were derived for each biopsy, including those taken prior to GSK2141795 treatment, using the improved Gene Set Enrichment z-score of Irizarry [29]. Briefly, this involves calculating t-statistics for differential expression of each protein in the signature in each sample, as compared with all other samples. A weighted average of these t-statistics has been shown to be normally distributed and thus can be used to derive a z-score for each sample, quantifying the degree of systematic up- or down-regulation of the set of proteins comprising the signature. Signatures were calculated for GSK2141795-upregulated and GSK2141795-downregulated proteins separately.

## SUPPLEMENTARY MATERIAL FIGURES AND TABLES










