# Supplementary material for: A putative biomarker signature for clinically effective AKT inhibition: correlation of in vitro, in vivo and clinical data identifies the importance of modulation of the mTORC1 pathway
Source: Oncotarget. 2015 Oct 19;6(39):41736–49. doi: 10.18632/oncotarget.6153 (PMC4747185; doi:10.18632/oncotarget.6153)
Supplement: Supplementary file 1 [file oncotarget-06-41736-s001.pdf]

A putative biomarker signature for clinically effective AKT inhibition: correlation of *in vitro*, *in vivo* and clinical data identifies the importance of modulation of the mTORC1 pathway

Supplementary Material

Cell Culture

Cells were maintained in RPMI 1640 (Sigma) supplemented with 10% foetal calf serum, penicillin, streptomycin, glutamine (Sigma), at 37°C, 5% CO_2 between passage numbers (20 and 30). For preparation of monolayers (2D) tumour cells were trypsinised from the monolayer culture, seeded into 96 well plates (approximately 5 x 10^3 cells per well) and incubated at 37°C, 5% CO_2 for 24 hours to allow attachment. For preparation of multi-tumour spheroids SKOV3 tumour cells were trypsinised from monolayer cultures and cell suspensions seeded in 24-well, 1% agarose-coated plates, approximately 10,000 cells per well. Spheroids were grown at 37°C, 5% CO_2 for 7 days. Apoptosis (caspaseGlo3/7, Promega) and cell viability (3-(4,5-Dimethylthiazol-2-yl)-2,5-diphenyltetrazolium bromide (MTT; Sigma)) assays were performed as described previously [11].

Pharmacokinetics

The in-life portion of this study was performed at the GSK Clinical Imaging Centre, UK, and the mass spectrometry (MS) analysis of this study was carried out by GSK, USA. All samples were stored at -80°C prior to analysis. Samples collected at the same nominal times were pooled for analysis. Prior to bio analysis, blood samples were mixed 1:1 (v:v) with HPLC grade water, and tissue samples were mixed 1:4 with HPLC grade water (w:v; tissue: water) and homogenised using a probe-type homogeniser. Mouse tissue homogenates, plasma and blood samples were analysed

for GSK2141795 using LC/MS/MS. Tissue homogenate concentrations (ng/mL homogenate) were converted to tumour tissue concentrations (ng/g tissue) by calculating the product of the measured homogenate concentration and the dilution factor incurred during sample processing. This conversion assumes complete extraction of the analyte from the tissue homogenate samples during bioanalytical sample preparation.

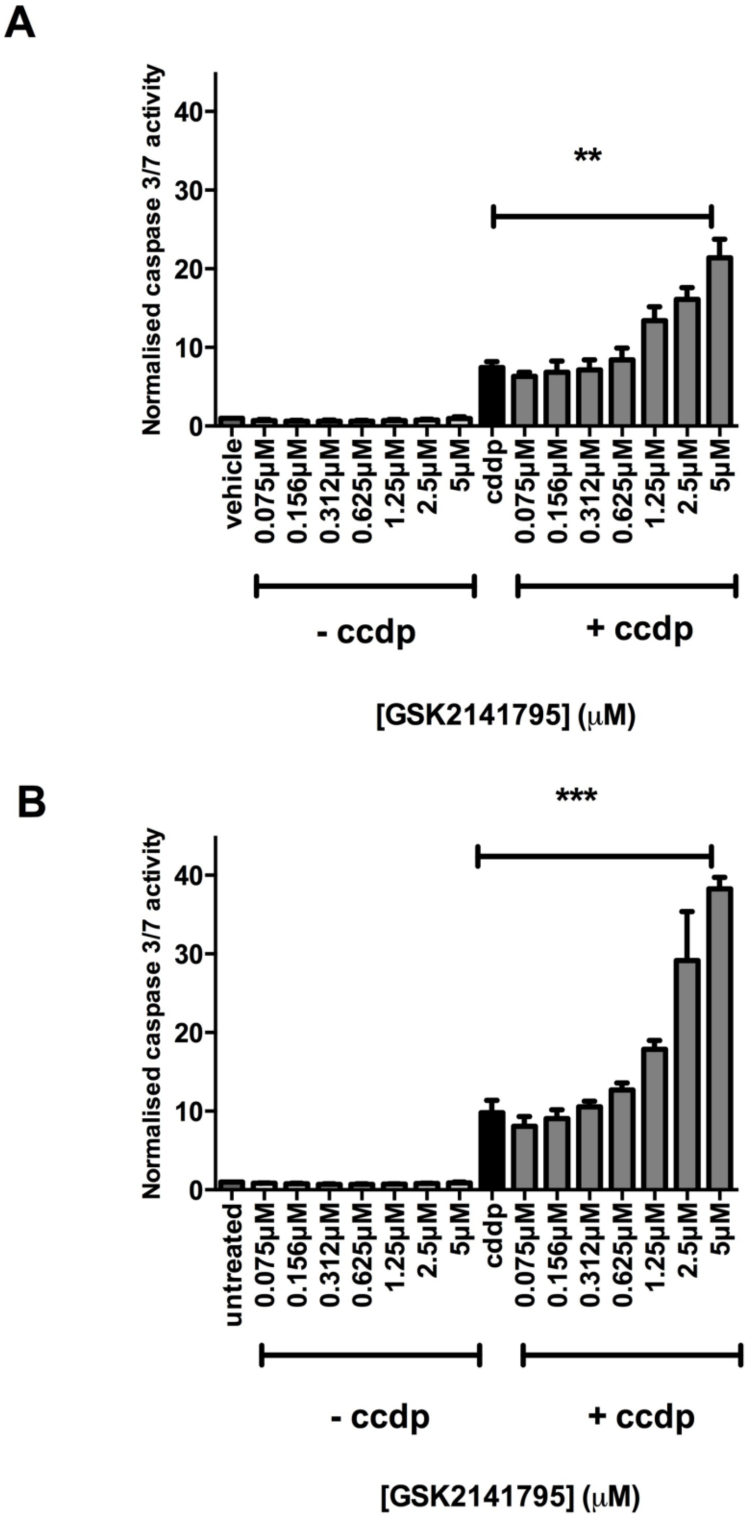

Figure S1: Caspase 3/7 activity and cell viability in SKOV3 monolayers exposed to GSK2141795 as a single agent or in combination with cisplatin (cddp; 25 µM) for 48 hours (A) and 72 hours (B). ***p*<0.01, ****p*<0.001 (unpaired t-test). Data shows mean of N=3 experiments performed in triplicates ± SEM.

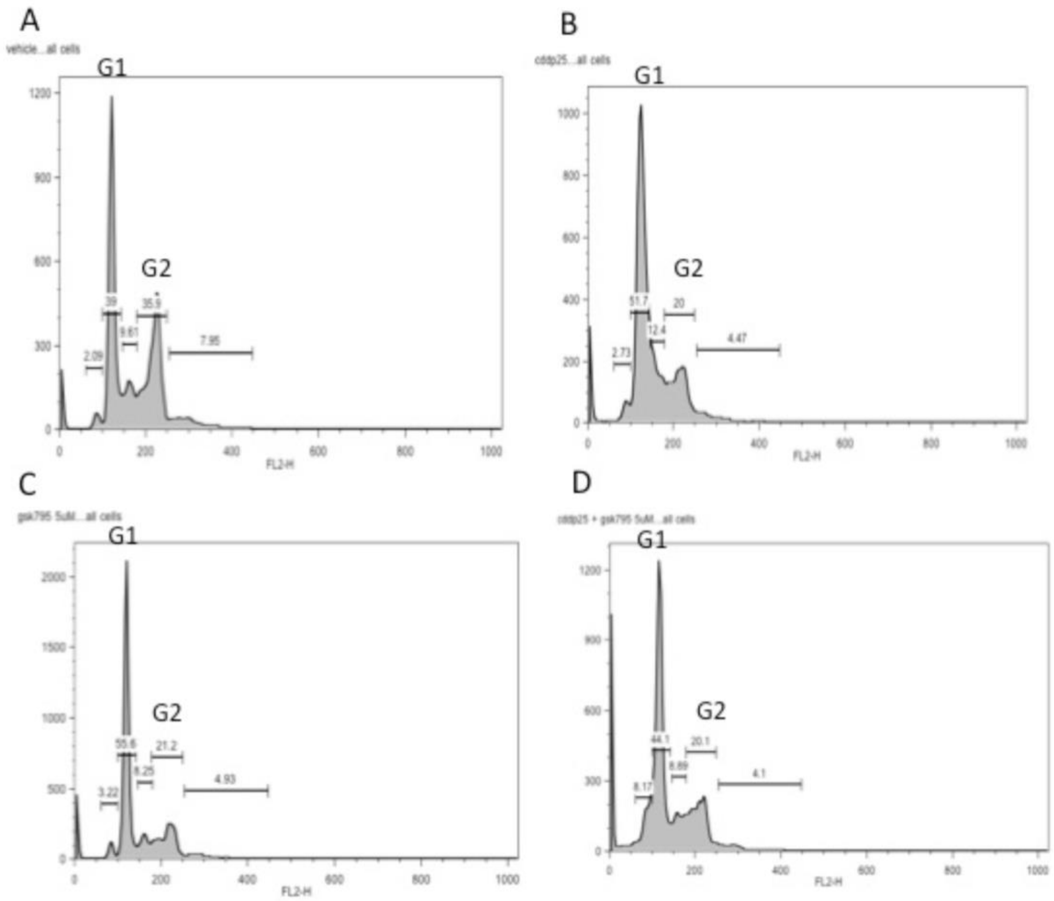

Figure S2: Cell cycle analysis of SKOV3 cells following treatment with (A) DMSO vehicle; (B) cisplatin (cddp; 25 µM); (C) GSK2141795 (5 µM); and (D) combination of GSK2141795 (5 µM) and cisplatin (25 µM). After 24 hours treatment with GSK2141795 as single agent or in combination with cisplatin, cells were labeled with propidium iodide (PI) and analysed by flow cytometry. Data shows the percentage of cell population in each phase of the cell cycle.

A

B

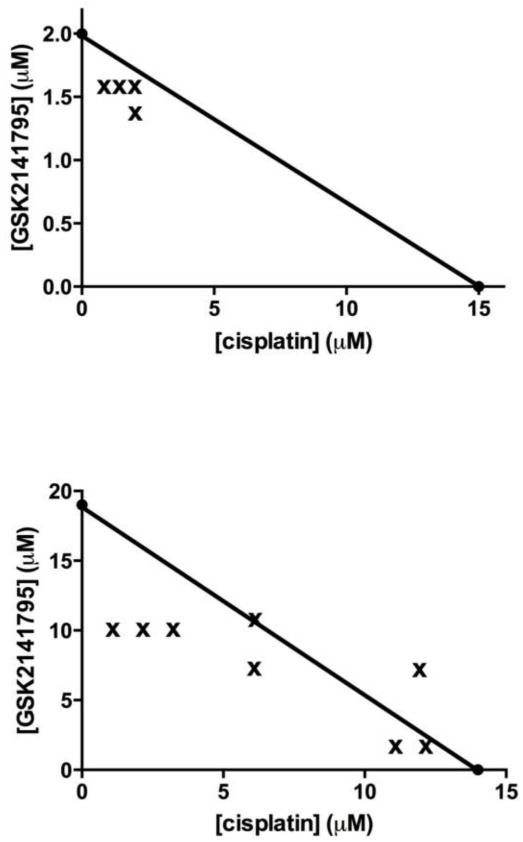

Figure S3: GSK2141795 and cisplatin interaction in SKOV3 (A) and PEO4 (B) cells. Isobologram generated from GSK2141795 and cisplatin IC_{50} values taken from the 2D monolayer studies. The IC_{50} values of combinations were determined with the 95% confidence limit and plotted. The points on the axes represent the IC_{50} values of either GSK2141795 or cisplatin as single agents. The line connecting the IC_{50} values represents the additivity line. Combination drug points occurring on or around the line represent additivity whereas points above the line represent antagonism. Points below the line represent synergy. All of The IC_{50} values of the GSK 2141795 and cisplatin combination in SKOV3 and most of the values in PEO4 were below the line suggesting synergy between these two compounds in these two cell lines.

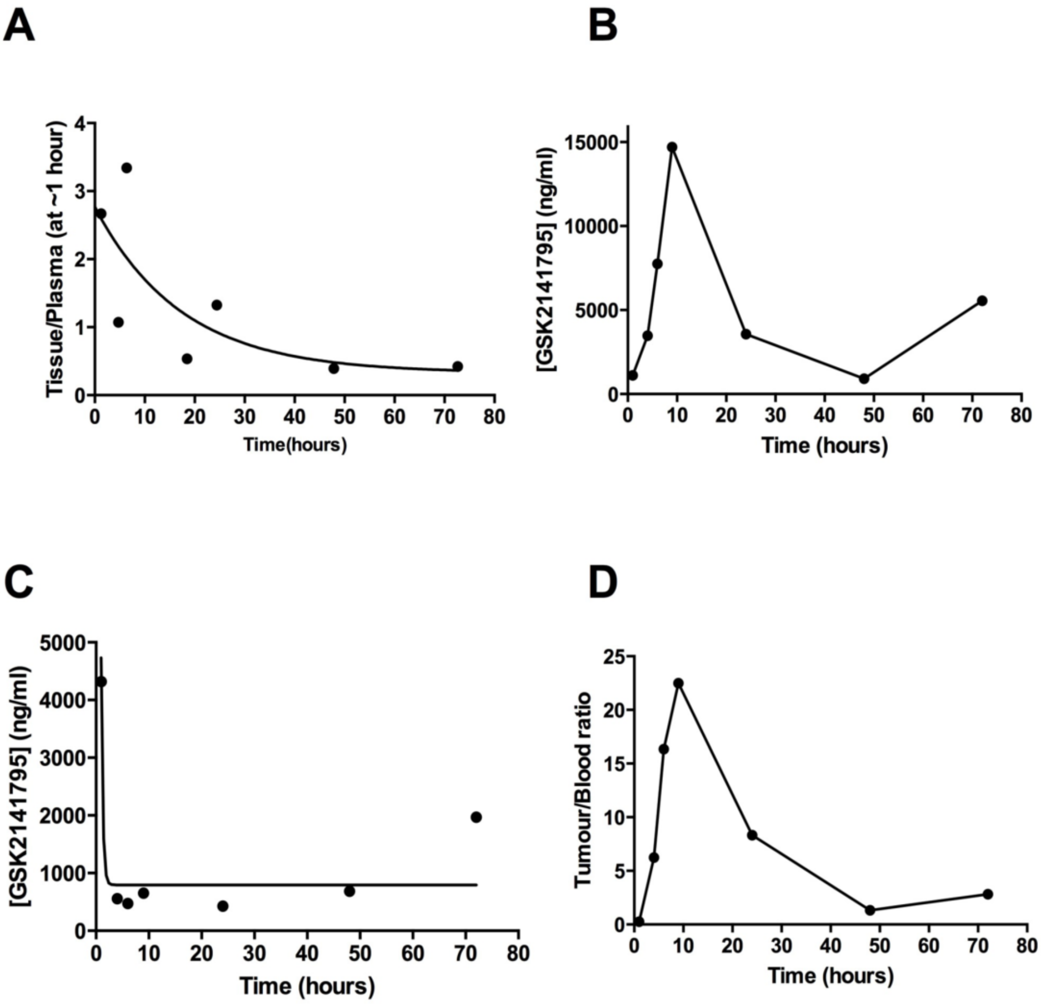

Figure S4: SKOV3 tumour bearing mice were treated with GSK2141795 for time ranging from 1 to 72 hours prior to [^{18}F]FDG administration. Uptake of [^{18}F]FDG into SKOV3 tumours in mouse xenografts decreased in a time-dependent fashion (t½ = 12 hours) following oral administration of 30mg/kg GSK2141795 (A). Pharmacokinetic data for GSK2141795 accumulating in xenograft tumours (B) and blood (C) up to 72 hours after a single oral administration at 30 mg/kg; with the subsequent ratio of tumour to blood (D) N=3 samples, pooled analysis.

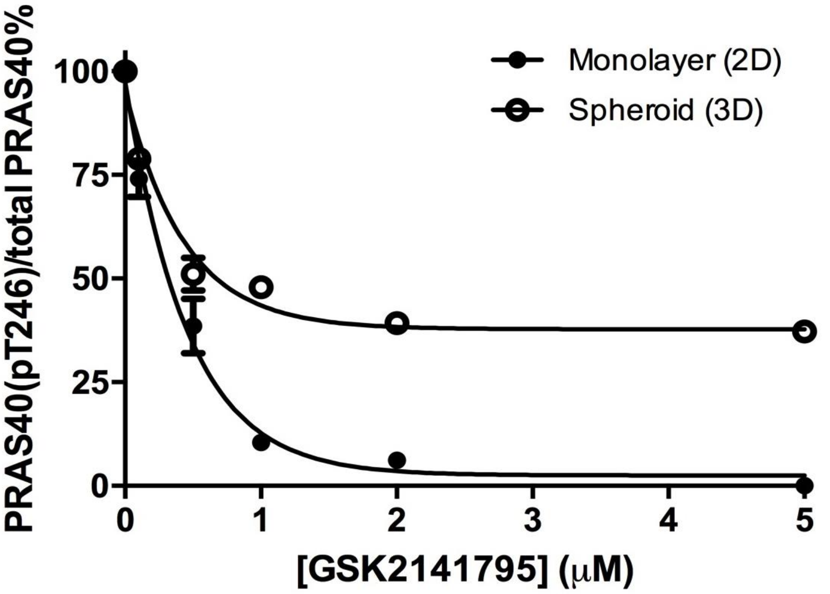

Figure S5: Inhibition of PRAS40 phosphorylation by GSK2141795 in SKOV3 monolayers and MTS. Protein concentration of phospho-PRAS40 (Thr246) and total PRAS40 was determined by enzyme-linked immunosorbent assay (ELISA) after 48hr treatment of SKOV3 cells with a range of concentrations of GSK2141795 (0.01 – 5 μM) in both monolayers and MTS. Data are the mean of N=2 experiments performed in triplicate ± SEM.
