# Supplementary material for: A putative biomarker signature for clinically effective AKT inhibition: correlation of in vitro, in vivo and clinical data identifies the importance of modulation of the mTORC1 pathway
Source: Oncotarget. 2015 Oct 19;6(39):41736–49. doi: 10.18632/oncotarget.6153 (PMC4747185; doi:10.18632/oncotarget.6153)
Supplement: Supplementary file 2 [file oncotarget-06-41736-s002.pdf]

Supplementary Table S1

Patient ID	Cancer type	FIGO stage	Grade	Histological subtype	GSK2141795 dose	Best CA125 response	CAS signal group
100	Ovarian	3c	3	Serous	50mg	-10	-
101	Ovarian	4	3	Serous	50mg	-36	+
102	Ovarian	4	2	Serous	50mg	-36	+
103	Ovarian	4	3	Endometrioid	50mg	13	-
1200	Ovarian	2a	3	Clear cell	75mg	-59	+
1201	Ovarian	3b	3	Serous	75mg	-76	+
1202	Ovarian	3c	3	Serous	75mg	-25	-
1203	Ovarian	4	1	Serous	75mg	15	-
1205	Ovarian	3c	3	Clear Cell	25mg 2wk->75mg 2wk	3	-
1206	Ovarian	3c	3	Serous	25mg 2wk->75mg 2wk	-8	-
1207	Endometrial	4b	3	Endometrioid	25mg 2wk->75mg 2wk	-33	+
1208	Ovarian	3	2	Serous	25mg 2wk->75mg 2wk	-71	+
